# Cremation

**Published:** 1875-06

**Authors:** 


The question of cremation, the burning of the dead, against inhumation or burying of the dead, has gone no short distance on its way to acceptance in Switzerland and Germany. At Zurich, where burial-ground is growing contracted, 2,000 persons have subscribed toward an association founded in favor of burning the dead. At Basle the movement has received the public approval of Orthodox clergymen—also on the ground of promotion of health in the community. In Germany the newspapers are talking about the subject a great deal, while one firm in Berlin has advertised the invention of a new furnace in which to perform the operation. And last, a church-warden of a Hebrew synagogue in the same city has proposed to establish on a new burial-ground, lately acquired, one of these furnaces.

---

The garden is woman's sphere, a natural theatre for her tastes, a remedy for half her ills. It is her academy, gymnasium, school of beauty. Here are the graces,—one with her rose in her hand, and another with her branch of myrtle. In their society she breathes the fragrant morning air, and rests at noon in the shade of the vine which her own fingers have trained.

---

Want less than you have, and you will always have more than you want.

